# Seasonal variation in calcium treatment after thyroidectomy as surrogate for post-operative hypocalcemia: a retrospective register-based national cohort study

**DOI:** 10.1186/s13044-022-00123-7

**Published:** 2022-03-19

**Authors:** Carl Kördel, Anna Koman, Robert Bränström, Adam Stenman

**Affiliations:** Department of Breast, Endocrine Tumors and Sarcoma, Karolinska University Hospital, Stockholm, Sweden and Department of Molecular Medicine and Surgery, Karolinska Institutet, 171 76 Stockholm, Sweden

**Keywords:** Hypocalcemia, Thyroidectomy, Seasonal variation, Vitamin D, Calcium

## Abstract

**Background:**

Hypocalcemia is one of the most common complications of thyroidectomy, and vitamin D deficiency has been found to be an independent risk factor. Sweden is located north of the 55th latitude, resulting in a significant seasonal variation in sun exposure, thereby large variation in the naturally occurring levels of vitamin D. This study aimed to determine if there is a correlation between season of surgery and post-thyroidectomy hypocalcemia.

**Methods:**

We conducted a retrospective register-based observation study on patients who had undergone total thyroidectomy during 2008–2015. In total, 7125 patients operated in Swedish facilities were identified via the Scandinavian Quality Register for Thyroid, Parathyroid, and Adrenal Surgery (SQRTPA). Patients operated during February–April were included in the dark group and patients operated during August–October were included in the bright group. Further stratification was made on the indication for surgery. The primary outcome was post-operative calcium treatment due to hypocalcemia, defined as having received calcium orally or intravenously before discharge.

**Results:**

The risk of receiving post-operative calcium treatment was significantly lower in the bright group (29.7%) compared to the dark group (35.1%), with a relative risk of 0.846 (*P <* 0.001). This correlation held true if the indication for surgery was goiter or thyrotoxicosis. For malignancy, there was no significant difference between the groups.

**Conclusion:**

In this cohort, total thyroidectomy performed during August–October was associated with a lower rate of calcium treatment given post-operatively when compared to total thyroidectomy performed during February–April. This would indicate a decreased risk of post-operative hypocalcemia if surgery was carried out after the brighter season.

## Introduction

Hypocalcemia is a common complication after total thyroidectomy [1, 2]. The rates vary greatly depending on several factors, including the definition of hypocalcemia, inclusion criteria and study population [3–6]. In a systematic review including 115 studies, transient and permanent hypocalcemia were found in 19–38% and 0–3%, respectively [6]. However, depending on the study population and definition, persistent hypocalcemia has been found to occur much more frequently [7]. Hypocalcemia after thyroidectomy is an effect of hypoparathyroidism occurring due to direct injury, devascularization, inadvertent excision of parathyroid glands, or mineral depletion of the bone caused by an increased metabolism [8, 9]. Besides female sex, higher age, Graves’ disease and parathyroid gland reimplantation, vitamin D deficiency has been found to be an independent indicator of transient post-operative hypocalcemia following thyroid surgery [4, 8, 10–12]. However, there is no clear cut-off value for vitamin D, and opinions diverge on to what extent vitamin D levels may predict post-operative hypocalcemia [6]. Previous research suggests that supplementing vitamin D in a population with widespread vitamin D deficiency may reduce the risk of hypocalcemia following thyroid surgery [13].

Exposure of the skin to solar ultraviolet B radiation is the major source of vitamin D, and only a tiny proportion is derived from dietary intake [14, 15]. Consequently, the risk of developing vitamin D deficiency is related to lack of exposure to sunlight, whatever the reason (e.g. season, altitude or skin cover) [16]. A prospective study of subtotal thyroidectomy performed on patients suffering from Graves’ disease suggested that there might be a correlation between hypocalcemia and surgery during the darker season, although significance was not obtained [17]. However, a group in Montreal showed a correlation between surgery during the winter and a higher risk for post-operative hypocalcemia [18]. This study included patients operated in Swedish facilities. Sweden is located north of the 55th latitude, resulting in significant seasonal variations in sun exposure. At 52 degrees north sun exposure is inadequate from October through March and no vitamin D3 will be produced during this time [15]. The naturally occurring vitamin D levels in European populations are highest at the end of summer and the beginning of fall. The lowest levels are found at the end of winter and the beginning of spring [19–22].

This study aims to determine whether the season of surgery does affect the risk of hypocalcemia after thyroidectomy in a Swedish setting. In order to do this, 7125 patients were identified via the Scandinavian Quality Register for Thyroid, Parathyroid, and Adrenal Surgery (SQRTPA). The hypothesis is that thyroidectomy during a season with higher naturally occurring vitamin D levels would have a protective effect on post-operative hypocalcemia, and therefore a lower rate of calcium treatment given post-operatively.

## Materials and methods

We performed a retrospective register-based cohort study on patients who had undergone total thyroidectomy from the 1st of January 2008 until the 31st of December 2015 in Sweden. The study population was extracted from SQRTPA. All patients who had undergone primary total thyroidectomy as well as completion after previous hemithyroidectomy were included. SQRTPA was established in 2004 to register thyroid, parathyroid, and adrenal surgery. The coverage of Swedish thyroid surgery in 2015 was 87%. However, this number is lower for some of the follow-up variables as presented below [23]. Data on patients operated during the period stated above was retrieved from the registry. These included age, sex, date of surgery, indication for surgery, type of surgery, oral and intravenous calcium treatment post-operatively due to hypocalcemia, prescribed supplement vitamin D due to hypoparathyroidism and calcium due to hypocalcemia at discharge, and at six-month follow-up. The phrasing in the registry forms regarding these treatments are “Hypocalcemia requiring treatment with iv calcium or oral calcium postoperatively”, and at the follow-up: “Calcium treatment due to hypocalcemia” and “Vitamin D treatment due to hypoparathyroidism”, meaning novel prescriptions for these indications. The primary outcome in this study was post-operative calcium treatment due to hypocalcemia, defined as having received calcium orally or intravenously before discharge. Secondary outcomes were the prescription of calcium and vitamin D supplements at discharge and six-month follow-up.

### Data collection and inclusion criteria

**Fig. 1:**
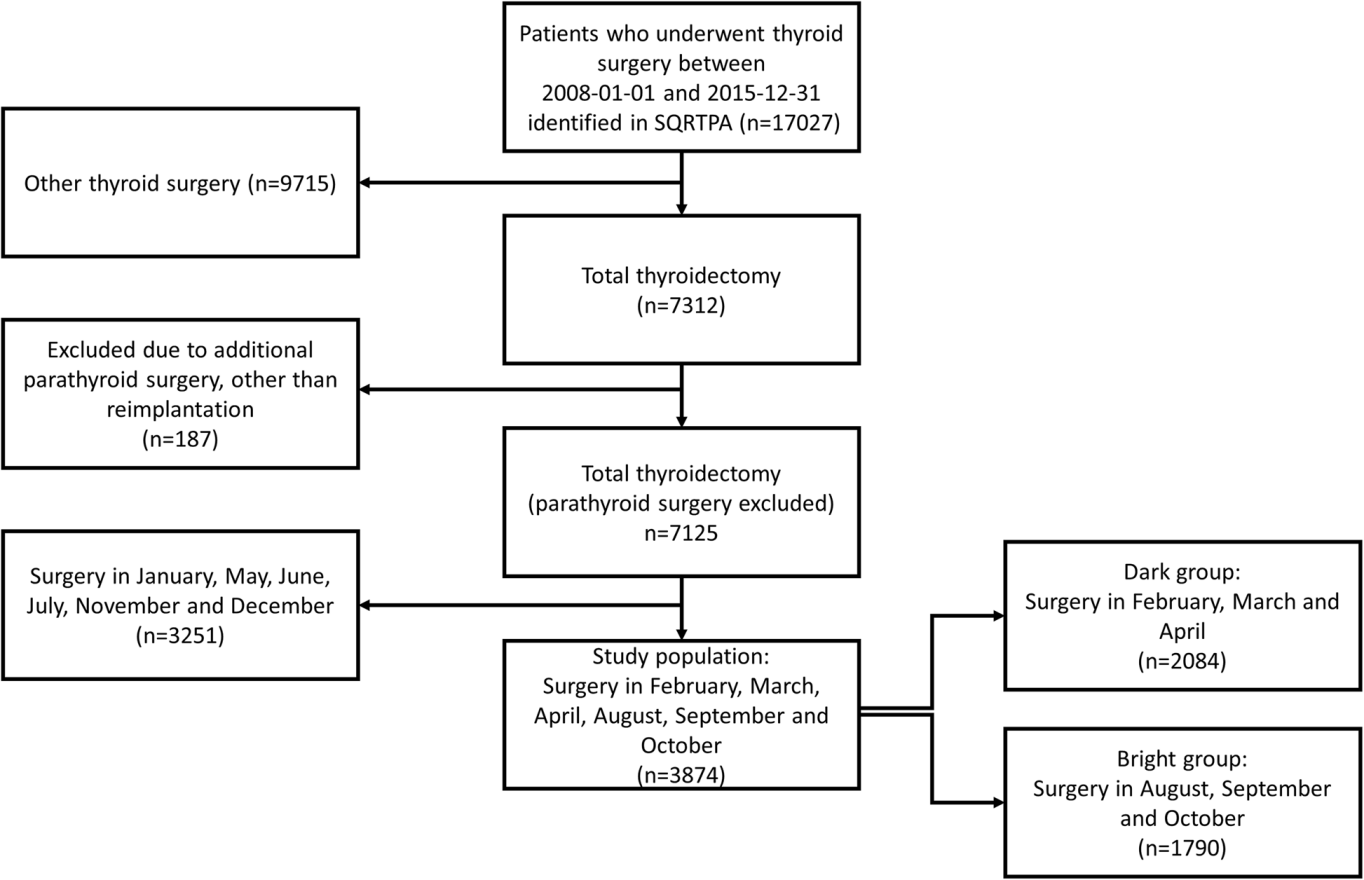
Flow chart of included patients. All patients who had undergone thyroid surgery during 2008–2015 were identified in SQRTPA. Out of these, all thyroidectomies were extracted and stratified on the season of surgery

A total of 17,027 patients were extracted from the SQRTPA. The analysis group is visualized in a flow chart in Fig. 1 and detailed in Table 1. Out of these, 7312 patients, who had all undergone total thyroidectomy, were identified. One hundred eighty-seven were excluded due to additional parathyroid surgery, other than reimplantation, leaving 7125 patients. All patients operated during February, March, and April were included in the dark group (*n* = 2084). All patients operated during August, September, and October were included in the bright group (*n* = 1790). The remaining patients (*n* = 3251) were excluded from further analyses. Surgery was performed at 38 units across Sweden, with the lowest frequency of 2 thyroidectomies and the highest of 1872 thyroidectomies over the study period.

**Table 1:** Patient characteristics. Presented in total and stratified by indication for surgery

	Dark group	Bright group	Total
**TOTAL**	2084	1790	3874
Sex, women, n (%)	1660 (79.7%)	1433 (80.1%)	3093 (79.8%)
Age, mean (range)	46.4	46.9	46.6 (0–94)
Parathyroid reimplantation, n (%)	548 (26.3%)	466 (26.0%)	1014 (26.2%)
**MALIGNANCY**	433	388	821
Sex, women n (%)	305 (70.4%)	256 (66.0%)	561 (68.3%)
Age, mean	50.9	53.4	52.0 (1–92)
Parathyroid reimplantation	171 (39.5%)	153 (39.4%)	324 (39.5%)
**GOITER**	578	471	1049
Sex, women n (%)	466 (80.6%)	388 (82.4%)	887 (81.4%)
Age, mean	54.8	55.0	54.9 (1–94)
Parathyroid reimplantation	110 (19.0%)	108 (22.9%)	218 (20.8%)
**THYROTOXICOSIS**	1062	923	1985
Sex, women n (%)	882 (83.1%)	784 (84.9%)	1692 (83.9%)
Age, mean	40.1	40.1	40.1 (0–88)
Parathyroid reimplantation	263 (24.8%)	205 (22.2%)	468 (23.6%)
**Missing data on indication**	11	8	19

Analyses were also carried out after stratifying on the main indication for surgery; malignancy (*n* = 821), goiter (*n* = 1049), and thyrotoxicosis (*n* = 1985). A small group of patients did not have their main indication registered (n = 19).

### Stratification

The levels of vitamin D in the study population were assumed to follow several previous studies conducted on European populations, showing the lowest levels of vitamin D at the end of winter and beginning of spring and the highest levels at the end of summer and beginning of fall [19–22]. Therefore, the population was stratified into two groups; the dark group and the bright group, with surgery performed in February, March, April or August, September, and October, respectively. The remaining patients were excluded from further analyses. In the next step, the population was stratified according to the main indication for surgery: malignancy (suspicion of malignancy, confirmed malignancy and completion thyroidectomy by malignancy), goiter (compression symptoms and recurrent cyst), and thyrotoxicosis.

### Statistics

All statistical analyses were performed using IBM SPSS Statistics, Version 26. Chi^2^-test was performed on categorical variables, including post-operative calcium treatment and prescribed supplements. An imputation analysis using linear regression was performed to reduce the bias that the missing data may provide regarding calcium supplements at six-month follow-up. Categorical variables were reported as numbers and percentages. Student’s *t* test was used on normally distributed continuous data and Mann-Whitney *U* test was used on skewed continuous data. A *P*-value of less than 0.05 was considered significant.

## Results

### Patient characteristics

The majority of the included patients, 79.8% (*n* = 3093), were women. Parathyroid reimplantation was performed in 26.2% (*n* = 1014) of the cases. The mean age was 46.6 years, ranging from 0 to 94. There were no significant differences between the dark and bright group in frequency of parathyroid reimplantation, sex or age for the total study population. However, when stratifying on the indication for surgery, the mean age differed to some extent between the dark and bright groups (50.9 and 53.4, respectively), (*P* = 0.056, Mann-Whitney *U* test) in the subgroup malignancy.

### Seasonal differences in post-operative calcium treatment before discharge

**Fig. 2:**
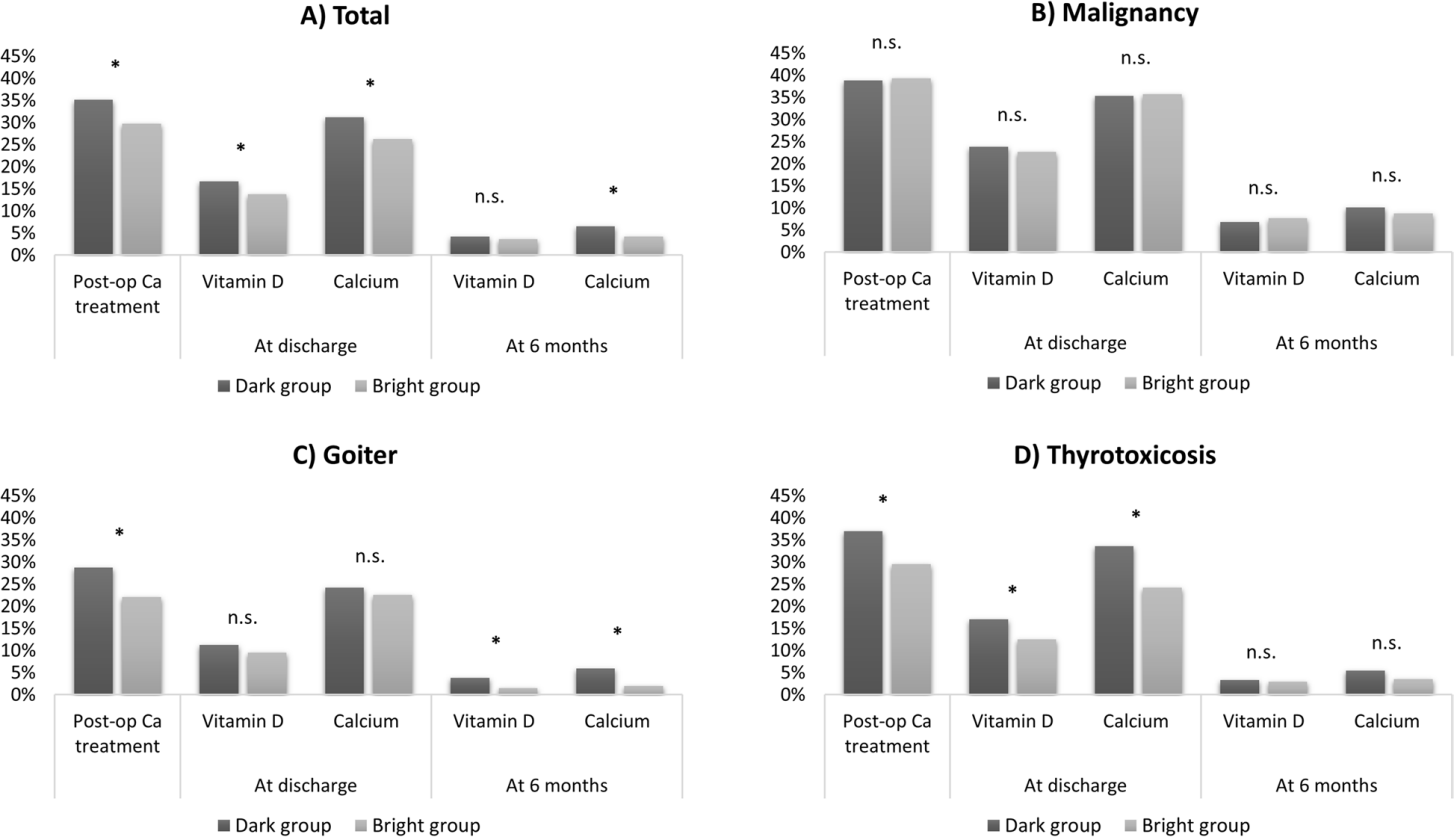
Rates of post-operative (post-op) calcium (Ca) treatment, rates of prescribed supplements at discharge and rates of prescribed supplements at six-months follow-up. Presented for the whole study population (**A**) and stratified by indication for surgery as malignancy (**B**), goiter (**C**) and thyrotoxicosis (**D**). * indicates *P* < 0.05

Out of all patients who underwent total thyroidectomy, post-operative calcium treatment was given in 2268/7108 cases (31.8%), missing data on 17 patients. The results are detailed in Table 2 and visualized in Fig. 2. In the final study population, surgery performed during February–April or August–October, post-operative calcium treatment was given in 1261/3870 cases (32.6%), missing data on 4 patients. The rate of patients receiving calcium treatment before discharge was significantly lower in the bright group, 530/1786 (29.7%), as compared to the dark group, 731/2084 (35.1%), (*P* < 0.001, Chi^2^-test). Thus, the relative risk of receiving post-operative calcium treatment was 15.4% lower in the bright group as compared to the dark group. When further stratifying the study population according to indication for surgery, this correlation was significant for both goiter and thyrotoxicosis with lower rates in the bright group (*P* = 0.015 and *P* < 0.001, Chi^2^-test). For malignancy, there was no significant difference between the groups, instead the rates of post-operative calcium treatment were found to be similar (*P* = 0.889).

**Table 2:** Hypocalcemia treated postoperatively and prescribed supplements. Rates of postoperative hypocalcemia, rates of prescribed supplements at discharge and rates of prescribed supplements at six-months follow-up. Presented for the whole study population and stratified by indication for surgery

TOTAL	Dark group	Bright group	***P***-value
**Hypocalcemia**			
Treated postoperatively	731/2084 (35.1%)	530/1786 (29.7%)	**< 0.001**
**Supplements prescribed at discharge**			
Vitamin D	349/2084 (16.7%)	247/1786 (13.8%)	**0.012**
Calcium	651/2084 (31.2%)	468/1786 (26.2%)	**< 0.001**
Both Calcium and Vitamin D	302/2084 (15.5%)	205/1786 (11,5%)	**0.006**
**Supplements prescribed at 6 month follow up**			
Vitamin D	78/1853 (4.2%)	56/1536 (3.6%)	0.352
Calcium	121/1853 (6.5%)	65/1536 (4.2%)	**0.003**
Both Calcium and Vitamin D	57/1838 (3,1%)	38/1530 (2,5%)	0.2811
**MALIGNANCY**			
**Hypocalcemia**			
Treated postoperatively	168/433 (38.8%)	152/387 (39.3%)	0.889
**Supplements prescribed at discharge**			
Vitamin D	103/433 (23.8%)	88/387 (22.7%)	0.723
Calcium	153/433 (35.3%)	138/387 (35.7%)	0.923
**Supplements prescribed at 6 month follow up**			
Vitamin D	27/396 (6.8%)	27/351 (7.7%)	0.645
Calcium	40/395 (10.1%)	30/344 (8.7%)	0.515
**GOITER**			
**Hypocalcemia**			
Treated postoperatively	166/578 (28.7%)	104 (22.1%)	**0.015**
**Supplements prescribed at discharge**			
Vitamin D	65/578 (11.2%)	43/470 (9.5%)	0.267
Calcium	140/578 (24.2%)	106/470 (22.6%)	0.526
**Supplements prescribed at 6 month follow up**			
Vitamin D	20/527 (3.8%)	6/413 (1.5%)	**0.030**
Calcium	31/522 (5.9%)	8/405 (2.0%)	**0.003**
**THYROTOXICOSIS**			
**Hypocalcemia**			
Treated postoperatively	393/1062 (37.0%)	272/921 (29.5%)	**< 0.001**
**Supplements prescribed at discharge**			
Vitamin D	181/1062 (17.0%)	115/921 (12.5%)	**0.005**
Calcium	357/1062 (33.6%)	223/921 (24.2%)	**< 0.001**
**Supplements prescribed at 6 month follow up**			
Vitamin D	31/930 (3.3%)	23/798 (2.9%)	0.591
Calcium	50/926 (5.4%)	27/780 (3.5%)	0.055

### Prescribed supplements at discharge and at six-month follow-up

Vitamin D supplements were prescribed at discharge at a significantly lower rate in the bright group (13.8%), as compared to the dark group (16.7%), (*P* = 0.012, Chi^2^-test), missing data on 4 patients. The rate of prescribed oral calcium supplements at discharge was also significantly lower in the bright group (26.2%), as compared to the dark group (31.2%), (*P* < 0.001, Chi^2^-test), missing data on 4 patients. Moreover, in the dark group, 15.5% (302/2084) were treated with both calcium and Vitamin D supplements, compared to 11.5% (205/1786) in the bright group. This comparison was found statistically significant (*P* = 0.006). The rate of prescribed oral calcium supplements at six-months follow-up was significantly lower in the bright group (4.2%) as compared to the dark group (6.5%), (*P* = 0.003, Chi^2^-test), missing data on 485 patients. An imputation analysis that is replacing these missing values with estimates, revealed significant correlations in 4/5 imputations (1: *P =* 0.021, 2: *P <* 0.001, 3: *P =* 0.091, 4: *P <* 0.001, 1: *P <* 0.001) which we interpret as satisfactory. At six-month follow-up, vitamin D supplements were prescribed to 3.6% of the patients in the bright group and 4.2% in the dark group (*P* = 0.352, Chi^2^-test), missing data on 442 patients. In the dark group, 3.1% (57/1838) of the patients were treated with both calcium and Vitamin D supplements, compared to 2.5% (38/1530) in the bright group (not significant). Rates of prescribed supplements in the subgroups are presented in Table 2 and Fig. 2.

## Discussion

This study calculated post-thyroidectomy calcium treatment rates to investigate a correlation between the season of surgery and post-thyroidectomy hypocalcemia. The overall rate of patients receiving post-operative calcium treatment before discharge, in the study population, was 32.6%. Given the wide range of rates of post-operative hypocalcemia reported in the literature, it is reasonable to say that this is in line with previously reported results [6].

It was found that post-operative calcium treatment was given significantly less frequently to patients operated at the end of summer and beginning of fall than patients operated at the end of winter and beginning of spring. The results obtained in this study supports findings previously presented by Mascarella et al. [18]. However, they used symptoms of hypocalcemia combined with plasma concentration of calcium instead of calcium treatment as their primary variable. On the other hand, the present study design made it possible to include a considerably larger sample size. Our studies also differed slightly in how the patients were stratified according to season.

In the next step, we aimed to investigate whether the previously discussed seasonal difference was persistent over time. Since neither calcium levels nor parathyroid hormone (PTH) are routinely measured on all patients after thyroidectomy at Swedish hospitals, prescription of vitamin D due to hypoparathyroidism and calcium supplements due to hypocalcemia, at discharge and at six-month follow-up were used as surrogate variables for persistent hypocalcemia. Calcium supplements were, in fact, prescribed more frequently to patients in the dark group at 6 months, as compared to the bright group. These rates are slightly higher compared to previously reported rates of persistent hypocalcemia [6]. The correlation was the same for vitamin D supplements; however, the difference was not statistically significant. This indicates that the observed seasonal difference in post-operative calcium treatment may also be true for persistent parathyroid failure. Since calcium supplement alone is rarely enough to correct plasma calcium levels when PTH is absent due to hypoparathyroidism, the patients need treatment with active Vitamin D. At follow-up, the surgeons are considering the registry wording “Vitamin D treatment due to hypoparathyroidism” which in the vast majority of cases means treatment with active Vitamin D. Moreover, since the treatment for hypocalcemia was not only temporary but prolonged at follow-up, this could be of importance in the clinical setting, since the patients then would be in need not only for surveillance of the levothyroxine substitutions but also of calcium medication, with possible impact on quality of life. Furthermore, data on prescribed supplements at the six-months follow-up were missing in 11% of the patients in the dark group and in 14% of the patients in the bright group, which may affect the result to some extent. However, we argue that this is a non-differential misclassification, and an imputation analysis providing the missing values with estimates, verified the results.

This retrospective register-based study does not provide any evident cause of the seasonal variation observed. Even though several factors could influence the correlation, we find it plausible that the naturally occurring vitamin D levels play an essential role. The present study was conducted on a large, nation-wide, study population. This large cohort made it possible to stratify on both season and indication for surgery with a sample size large enough in every subgroup. The study population was divided into groups based on the expected highest and lowest vitamin D levels. To verify that the levels of vitamin D are, in fact, responsible for the differences between the dark and the bright group, it would be necessary to correlate actual vitamin D values to the risk for post-operative hypocalcemia. This has previously been done, but not to our knowledge correlated to season [4, 10].

The seasonal difference in the rate of post-operative calcium treatment was still seen after stratifying on indications for surgery, both goiter and thyrotoxicosis. Interestingly, no seasonal difference was found if the indication for surgery was confirmed malignancy or suspicion of malignancy. One can speculate that the selection of cancer patients may influence this to specific units and/or surgeons. Furthermore, the difference in the biological nature of the various indications for surgery may affect calcium metabolism. In a previously published prospective observational study “hungry bone syndrome” caused by mineral depletion of bone due to increased bone metabolism during the period of thyrotoxicosis was found to be the major cause of hypocalcemia after total thyroidectomy [9]. The fact that no seasonal variation was seen if the indication for surgery was malignancy may be influenced by the unequal distribution of age, since high age is associated with a higher risk of post-operative hypocalcemia [12]. Although not statistically significant, mean age was slightly higher in the bright group which could potentially mask the seasonal difference seen in the subgroups.

One can argue that the seasonal variation in calcium treatment may be influenced by different levels of surgical skill over the year or that more difficult cases are prioritized during the summer. However, in Sweden thyroid surgery is only performed by specialized ENT- and endocrine surgeons irrespective of the time of the year, and medical staffing (physicians, anesthesiologists and operating assistants) is largely the same throughout the year, as a structural bias as a result of this can probably not explain seasonal differences. Furthermore, a predominance of more difficult cases during the summer could lead to higher rates of post-operative hypocalcemia and, if anything, diminish the observed seasonal variation.

As mentioned above, actual Ca^2+^ values were not used to measure hypocalcemia, which may have been intuitive. This was because analysis methods and cut-of values may differ over time and between laboratories; this definition was not considered suitable for this study set up. It was also noted that Ca^2+^ concentrations were not documented for a large proportion of the study population. Consequently, it was decided to use post-operative calcium treatment as the primary variable. A potential advantage of this definition is that it might include patients with symptoms of hypocalcemia but with Ca^2+^ in the lower normal range of reference. Of course, this makes it possible to let subjective decisions and the surgeon’s preference influence the results. However, national guidelines on handling post-operative hypocalcemia has been developed to reduce the risk of patients being treated differently depending on operating units and individual surgeons. Furthermore, the large sample size in this study could probably compensate for differences of this nature.

## Conclusion

In conclusion, within the context of these limitations, our study shows that the rate of post-operative calcium treatment was significantly lower in patients undergoing surgery during the months following the brighter season. This correlation was significant if the indication for surgery was goiter or thyrotoxicosis, but not malignancy. This indicates a decreased risk of post-operative hypocalcemia if surgery is carried out after the brighter season. It is plausible that the naturally occurring vitamin D levels play a role in this seasonal difference; however, further research is needed to verify this. Taken together, the findings of this study may help to identify patients at risk of developing hypocalcemia, and also support the idea that preoperative medication with vitamin D in a selection of patients could reduce the risk of hypocalcemia post thyroidectomy.

## Data Availability

The dataset generated and analyzed during the current study is available from the corresponding author on reasonable request and upon approval from SQRTPA.

## Abbreviations

SQRTPA: The Scandinavian Quality Register for Thyroid, Parathyroid, and Adrenal Surgery
Ca^2+^: Ionized calcium
PTH: Parathyroid hormone
